# Analysis of the Oral Microbiome in a Patient with Cardiofaciocutaneous Syndrome and Severe Periodontal Disease: Impact of Systemic Antibiotic Therapy

**DOI:** 10.3390/antibiotics11121754

**Published:** 2022-12-04

**Authors:** Carolina Muñoz Navarro, María del Carmen Sánchez Beltrán, Carolina Arriagada Vargas, Pilar Batalla Vázquez, Márcio Diniz Freitas, Jacobo Limeres Posse, Pedro Diz Dios, Eliane García Mato

**Affiliations:** 1 Special Care Dentistry Postgraduate Training Program, School of Medicine and Dentistry, University of Santiago de Compostela (USC), 15782 A Coruña, Spain; 2 GINTRAMIS Research Group (Translational Research Group on Microbiota and Health), Department of Medicine, Faculty of Medicine, University Complutense, 28040 Madrid, Spain; 3 Medical-Surgical Dentistry Research Group (OMEQUI), Health Research Institute of Santiago de Compostela (IDIS), University of Santiago de Compostela (USC), 15782 A Coruña, Spain

**Keywords:** cardiofaciocutaneous syndrome, periodontitis, antibiotics, microbiome

## Abstract

An 8-year-old girl diagnosed with cardiofaciocutaneous syndrome presented to our department with gingival pain, inflammation, and bleeding. Her medical history included hypoplasia of the corpus callosum, intellectual disability, trichothiodystrophy, global developmental delay, myopia, laryngomalacia, hypothyroidism, and osteoporosis. A diagnosis was reached of “periodontitis as a direct manifestation of systemic diseases”. During 9 years of follow-up, there were exacerbation episodes with spontaneous gum bleeding, ulcers in the interdental papilla, tooth mobility, and progressive tooth loss. Some of these exacerbation episodes resolved clinically with the administration of amoxicillin and metronidazole. We therefore proposed an oral microbiome study (subgingival and saliva samples) before and after antibiotic therapy. The most abundant genera at the subgingival level before administering antibiotics were *Prevotella*, *Streptococcus*, *Fusobacterium*, *Leptotrichia,* and *Aggregatibacter*. Of the 94 genera sequenced, 57 were less abundant in the post-treatment state than at baseline, particularly certain Gram-negative periodontal pathogens such as *Porphyromonas, Treponema, Aggregatibacter, Fusobacterium,* and *Campylobacter*. In contrast, other genera related to oral health, such as *Haemophilus, Granulicatella,* and *Abiotrophia*, showed an increase after administering the antibiotic. In conclusion, periodontitis exacerbations as a direct manifestation of systemic disease can occasionally be controlled exclusively with systemic antibiotics, without the need for performing mechanical periodontal therapy. This clinical recovery is correlated to substantial changes in the oral microbiome, which lead to the recovery of eubiosis of the microbiota.

## 1. Introduction

Periodontitis is a highly prevalent disease of inflammatory-infectious etiology that affects tooth-supporting tissues [1]. The disease’s etiopathogenesis is attributed to an imbalance between the subgingival biofilm and the host’s immune response [1]. The global age-standardized prevalence of periodontitis is estimated at approximately 10% [2] but can exceed 40% among those older than 40 years [3].

Although the conventional treatment of chronic periodontitis is based on scaling and root planning of the diseased periodontal pockets (with flap surgery in severe cases), it has been suggested that the adjuvant administration of systemic antibiotics provides a significant improvement in the clinical parameters [4], even in patients with aggressive periodontitis [5]. The most widely used antibiotics in clinical studies have been penicillins, tetracyclines, macrolides, quinolones, and nitroimidazoles, alone or in combination [6].

In 2017, the American Academy of Periodontology and the European Federation of Periodontology proposed a new classification of periodontal and peri-implant diseases, which establishes three categories of periodontitis: necrotizing periodontal diseases, periodontitis itself, and periodontitis as a manifestation of systemic diseases. In this last category, periodontitis develops as a consequence of certain syndromes or states of immunosuppression, which create increased susceptibility to periodontal disease [7,8].

We describe a case of a girl diagnosed with cardiofaciocutaneous syndrome (CFCS), a congenital disease with a low prevalence, characterized by craniofacial dysmorphism, cardiac malformations, ectodermal abnormalities, neuromotor retardation, and intellectual disability [9]. CFCS belongs to the group of RASopathies, a group of genetic diseases that share modulation inside the MAPKinase pathway [9], which present characteristic dysmorphic facial findings [10], including type 1 neurofibromatosis, Noonan syndrome, Costello syndrome, multiple lentigines, loose anagen hair syndrome and Legius syndrome [11]. The patient presented with periodontal disease presumably as a “manifestation of systemic disease”. In this study, we present the analysis of the patient’s oral microbiome to demonstrate the clinical–microbiological correlation of their response to antibiotic therapy.

## 2. Materials and Methods

### 2.1. Case Report

An 8-year-old girl diagnosed with CFCS (mutation in heterozygosity in the MAP2K1 gene) was brought for consultation to the Special Care Dentistry Unit (University of Santiago de Compostela, Spain) with a chief complaint of oral pain associated with inflammation and spontaneous gingival bleeding.

Her medical history included the following: hypoplasia of the corpus callosum, temporal leptomeningeal cysts, intellectual disability, severe myopia, language retardation, grade 2 laryngomalacia, weight-height growth delay (failure to thrive), kyphosis, pectus excavatum, hip dysplasia, osteoporosis, hypothyroidism, iron deficiency and recurrent episodes of erythrokeratoderma. The craniofacial examination revealed a syndromic phenotype, with trichothiodystrophy (scarce and fragile hair due to low sulfur content), loss of eyebrow tail (Hertoghe’s sign), hypertelorism, wide-based nose, and low implanted ears. The patient was undergoing long-term therapy with levothyroxine and a retinoid (acitretin).

The patient had prematurely lost the temporary molars and presented an abnormal eruption pattern (upper premolars completely erupted), deep caries in the first lower molars, and generalized accumulation of dental plaque due to poor oral hygiene. The gums were significantly inflamed, with bleeding gums in isolated areas, which increased after probing, with diseased periodontal pockets in 51% of the probed points (particularly on the lingual and palatal tooth surfaces). We established a diagnosis of “periodontal disease as the direct manifestation of systemic disease” and started treatment with topical antiseptic (chlorhexidine), systemic antibiotic therapy (amoxicillin and metronidazole), and non-surgical periodontal treatment (scaling and root planning).

**Figure 1:**
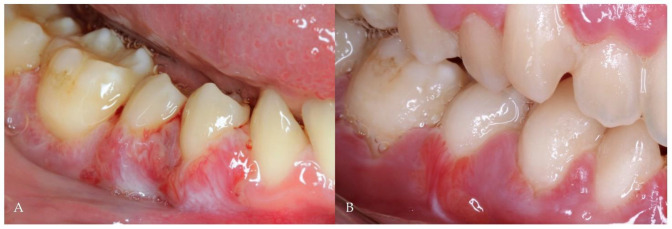
(**A**) Periodontal disease exacerbation; (**B**) Clinical outcome after one week of antibiotic therapy (without mechanical treatment).

**Figure 2:**
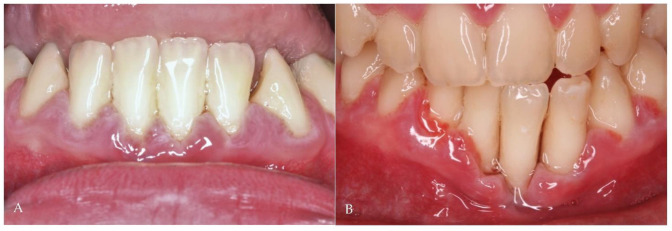
(**A**) Periodontal disease involving anterior teeth; (**B**) Periodontal disease progression despite periodic mechanical treatment (3 years later).

During the follow-up period (9 years), periodontal maintenance sessions were performed 3 times a year, which consisted of tartar removal with ultrasound and scaling and root planning in the more compromised areas (on 5 occasions, the procedure was performed on all teeth). During these years, the patient had recurrent episodes of exacerbation (Figure 1A) with pain, spontaneous gingival bleeding, ulcers in the interdental papilla, and increased tooth mobility, which were resolved clinically with the administration of systemic antibiotics without the need for mechanical periodontal procedures (Figure 1B). Despite the periodic periodontal treatment, the periodontal pockets progressively deepened and bone loss increased with time (Figure 2A,B), causing the premature loss of some teeth. However, the favorable clinical response of the recurrent episodes of periodontitis exacerbation to the systemic antibiotic therapy was the main argument for establishing the analysis of its impact on the composition of the oral microbiota.

### 2.2. Sampling and Extraction of Bacterial DNA

Unstimulated saliva samples were collected using a spitting method in which the participant was instructed to swallow before starting to collect saliva. The participant then accumulated saliva behind closed lips and spat it out into an assay tube at the end of each minute for a total of 3 min, as has previously been described [12].

Subgingival samples were collected from 4 locations by quadrant, specifically from those that had increased pocket depth with bleeding when probing [13]. The sampling was performed after isolating the area using cotton rolls and removing the supragingival biofilm. Two medium-sized tips of sterile paper (Maillefer, Ballaigues, Switzerland) were then inserted consecutively in each selected location, keeping them as subgingival as possible for 10 s [13]. The 8 paper tips were then pooled into one vial containing 1.5 mL of molecular water.

Both types of samples collected, saliva and subgingival samples, were immediately transported to the analysis laboratory, with no more than 2 h having elapsed between collection and shipment to the laboratory for analysis. Once the samples were received, they were processed immediately. The total DNA of the saliva and subgingival samples was extracted using a commercial kit (MolYsis Complete 5, Molzym Gmbh & Co. KG. Bremen, Germany) following the manufacturer’s instructions (according to the protocol for extracting bacterial DNA from step 6, skipping the preliminary phases). The extracted DNA was eluted in 100 µL of sterile distilled water (Roche Diagnostic GmbH, Mannheim, Germany) and frozen at −20 °C until its analysis in the Foundation for Health Promotion and Biomedicine (Fundación para la Promoción de la Salud y Biomedicina, FISABIO; Valencia, Spain).

Subsequently, the patient was prescribed antibiotic therapy with amoxicillin (40 mg/kg/day) and metronidazole (30 mg/kg/day) for 7 days. Upon completing the antibiotic therapy, new saliva samples were collected and subgingival samples were taken from the same 4 locations by quadrant; the sampling and DNA extraction was repeated applying the same methodology.

### 2.3. Gene Sequencing of rRNA 16S with Illumina Sequencing

The composition and structure of the samples’ microbial communities were assessed using a marker-based approach, using the amplification and sequencing of the variable regions V3–V4 of gene rRNA 16S [14]. We employed MiSeq technology (Illumina, San Diego, CA, USA) and included a negative control for the DNA extraction. After the genetic amplification of 16S rDNA, the multiplexing step was performed by preparing the DNA library with the Nextera XT Kit (FC-131-1096; Illumina). The sequencing of the libraries was performed using a paired-end experiment of 2300 bp (MiSeq v3 Reagent Kit [MS-102-3001]; Illumina) in a MiSeq sequencer according to the manufacturer’s instructions (Illumina). The quality was evaluated using the prinseq-lite program [15]. The data were obtained using an ad hoc pipeline written in the R Statistics Environment [16], making use of several open-source libraries. This approach allows us to describe and quantify the alpha and beta microbial diversity and study the taxonomic profiles from the phylum to the species level.

### 2.4. Taxonomic Assignment

We analyzed the sequencing data obtained by QIIME2 [17]. To this end, we eliminated the noise in the chimeric sequences and the short readings and those of low quality; we also trimmed the paired-end primers, using the DADA2 pipeline [18]. The data representation in the Krona graphs was performed using the Krona hierarchical browser program [19].

### 2.5. Statistical and Bioinformatic Analysis

The metataxonomic analysis was conducted on a total of 4 samples, one saliva sample and one subgingival plaque sample at baseline (compatible with an episode of periodontitis exacerbation) and the other two (saliva and subgingival plaque) after completing the antibiotic therapy. Due to the lack of copies and the absence of rankings to accommodate the data, we were unable to calculate the differences in the bacterial abundance between the groups of samples using the Wilcoxon rank sum test and the Kruskal–Wallis index between the several metadata factors.

For the diversity and significance analysis, we performed the statistical analysis using the R Statistics environment [16]. We analyzed the depth of the sequencing of the samples using the rarefaction curves. We also estimated the main bacteria and the microbiota’s taxonomic composition in each sample. Applying the phyloseq R package, we employed 4 metrics not based on the phylogeny, namely, species observed, Chao1metric, abundance-based coverage estimator (ACE), and the Shannon index to assess the alpha diversity, which represents the diversity contained within the communities. We applied a principal coordinates analysis (PCoA) with weighted UniFrac distances to assess the beta diversity, which represents the diversity shared between communities.

## 3. Results

### 3.1. Subgingival Samples

**Figure 3:**
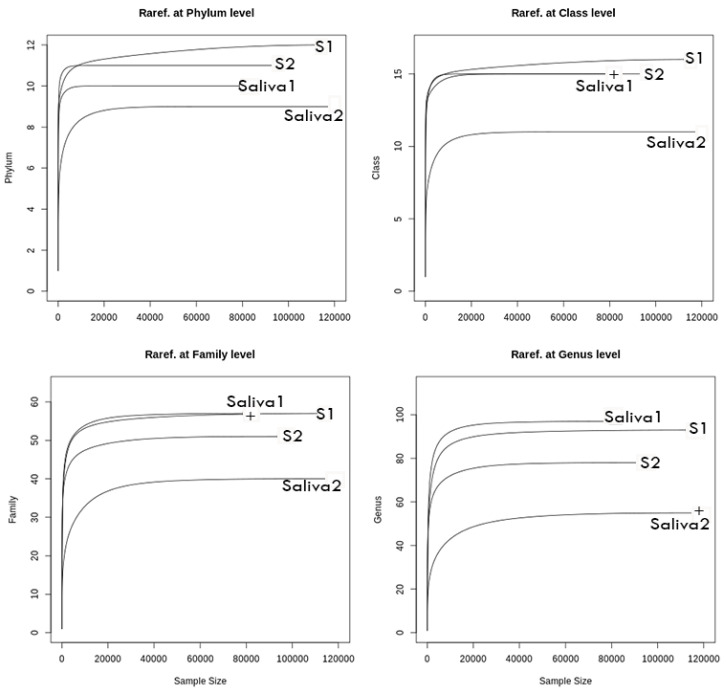
General rarefaction graphs. Rarefaction analysis permits estimating the overall diversity covered by the obtained squences. The figures represent the number of sequences (x axis) versus the number of taxa (y axis). S1, baseline subgingival sample; S2, post-therapy subgingival sample; Saliva 1, baseline saliva sample; Saliva 2, post-therapy saliva sample.

In the subgingival samples, we obtained a mean of 114,602 high-quality readings at baseline and 95,663 readings in the post-therapy situation, allowing for a complete description of the bacterial diversity in both conditions (Figure 3). The amplicon sequence variants (ASVs)) were assigned to 11 phyla, 16 classes, 33 orders, 55 families, 94 genera, and 197 species. When comparing the two conditions and representing the number of sequences read versus the number of different taxa identified, the resulting rarefaction curves showed taxonomic differences between the two conditions, mainly at the genus level (Figure 3), with a relevant reduction in bacterial diversity in the post-therapy situation compared with baseline.

**Figure 4:**
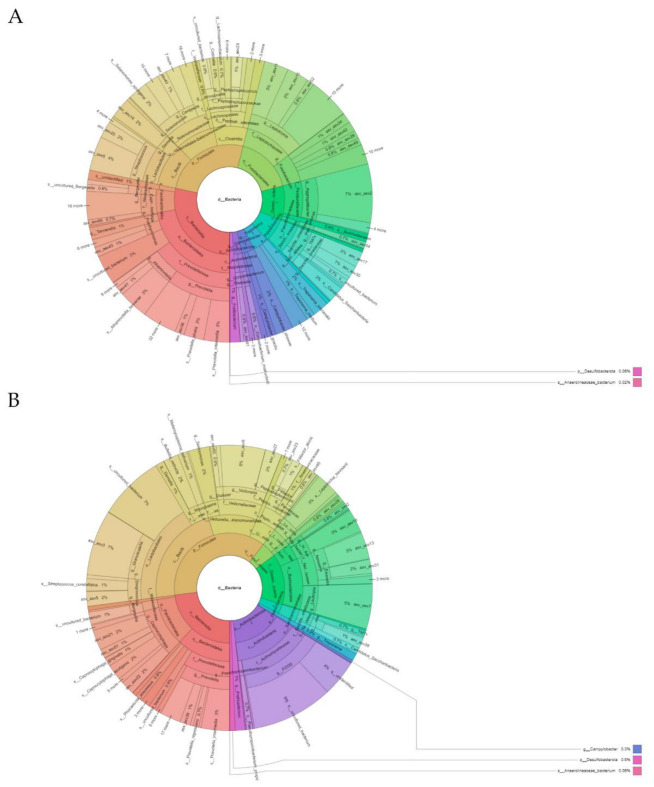
Representation created using the Krona hierarchical browser of the phylotypes identified at the phylum, class, order, family, genus, and species level in an individual with periodontitis: (**A**) Baseline subgingival sample; (**B**) Subgingival sample compatible with gingival health after antibiotic therapy.

The subgingival microbiota observed at baseline (before the antibiotic therapy) revealed a total of 11 phyla, 15 classes, 32 orders, 54 families, 90 genera, and 183 species. The microbiota were dominated (≥5% of relative abundance) at the phylum level by *Bacteroidota* (28.33%), *Firmicutes* (25.64%), *Fusobacteriota* (16.99%), *Proteobacteria* (10.06%), and *Patescibacteria* (7.00%) (Figure 2A and Table S1). The most abundant genera (≥5% of relative abundance) were *Prevotella* (10.12%), *Streptococcus* (6.36%), *Fusobacteria* (6.23%), *Leptotrichia* (10.70%) and *Aggregatibacter* (7.82%) (Figure 4A and Table S2).

After administering the antibiotic, the subgingival microbiota showed a change in pattern with respect to the relative bacterial abundance. Although we detected a total of 11 phyla, 15 classes, 29 orders, 58 families, 72 genera, and 139 species, the microbiota were dominated in this situation (≥5% of relative abundance) at the phylum level by *Firmicutes* (37.53%), *Bacteroidota* (22.95%), *Proteobacteria* (14.95%), and *Actinobacteriota* (14.45%) (Figure 4B and Table S1). The most abundant genera (≥5% of relative abundance) corresponded to *Actinomyces* (13.34%), *Prevotella* (7.98%), *Abiotrophia* (7.44%), *Granulicatella* (7.42%), *Veillonella* (6.99%), *Capnocytophaga* (8.92%), and *Lautropia* (5.11%) (Figure 4B and Table S2).

As shown in Table S2, 57 of the 94 genera sequenced were less abundant after the therapy than in pretreatment, highlighting a reduction in some of the main Gram-negative periodontal pathogens such as *Porphyromonas*, *Treponema*, *Aggregatibacter*, *Fusobacterium* and *Campylobacter*, along with other subgingival pathogens such as TM7x, *Pseudomonas* and *Alloprevotella*. In contrast, other oral health-related genera such as *Haemophilus*, *Granulicatella,* and *Abiotrophia* showed an increase in their relative abundance post-therapy.

In terms of relative abundance of the phylum *Actinobacteriota* following antibiotic therapy, of the 7 phylotypes identified, the genera *Corynebacterium* and *Olsenella* decreased by 94.0% and 91.0%, respectively. In contrast, the abundance of *Actinomycetes* of the genus F0332 and *Pseudopropionibacterium* increased by more than 97.1% (Table S2).

Of the 14 identified phylotypes of the phylum *Bacteroidota*, there was an 80.6% reduction in the relative abundance of the genus *Porphyromonas*, a 94% reduction for *Alloprevotella,* and a 62.6% reduction for *Tannerella* after administering the antibiotic. The genus *Chryseobacterium* was detected exclusively post-therapy (Table S2). In other genera, an increase was detected in their relative abundance, highlighting *Rikenellaceae_RC9_gut_group* and *Capnocytophaga* (87.6% and 56.5%, respectively).

In the phylum *Campylobacterota*, the only ASV detected corresponded to the genus *Campylobacter*, which decreased its abundance by 91.6% after the antibiotic therapy.

Of the 2 identified ASVs belonging to the phylum *Desulfobacterota*, the genus *Desulfobulbus* was detected exclusively after the antibiotic therapy, and an 88.2% increase was confirmed in the genus *Desulfovibrio* post-therapy (Table S2).

*Firmicutes* was the most abundant phylum of the ASVs at the subgingival level, including a total of 42 phylotypes. The genera with a presence greater than 1% that were noteworthy due to the reduction of their relative abundance after the antibiotic therapy were *Catonella*, *Centipeda,* and *Johnsonella* (96.1%, 95.8%, and 87.3% reduction, respectively), most especially *Peptococcus* (99.5%). In the post-therapy situation, neither the genus *Clostridia UCG-014* nor the genus *Peptoanaerobacter* were detected. In contrast, after administering the antibiotic, there was an increase in the presence of the genera *Filifactor* (84.0%), *Veillonella* (95.7%), *Builleidia* (97.7%), *Granulicatella* (98.3%), *Mycoplasma* (98.6%), and *Abiotrophia* (99.3%) (Table S2).

The phyla *Fusobacteriota* and *Patescibacteria*, considered the most abundant at baseline, ceased to be so after the treatment (Table S2). In the phylum *Fusobacteriota*, which included three phylotypes, *Fusobacterium* was the genus with the greatest reduction in relative abundance post-therapy (80.5%). In the phylum *Patescibacteria* (6 phylotypes), the genus *TM7x* also decreased by approximately 80%, and the genera *Streptobacillus, Absconditabacteriales_SR1,* and *Saccharimonas* were undetectable.

The antibiotic therapy resulted in an increased relative abundance of various genera of the phylum *Proteobacteria*, represented by 16 phylotypes. The Gamma-proteobacterias *Haemophilus* (99.4%), *Lautropia* (99.16%), *Eikenellaun* (97.2%), and *Neisseria* (86.2%) increased their presence. We also detected the genus *Kingella*, which was undetectable at baseline. In contrast, we observed a reduced abundance after treatment of the genera *Aggregatibacter* (89.5%), *Pseudomonas* (90.5%), and *Cardiobacterium* (91.5%), and the genera *Vibrio*, *Acinetobacter,* and *Moraxella* became undetectable (Table S2).

The abundance of the genus *Treponema*, the only representative of the phylum *Spirochaetota*, reduced by 81.5% after the antibiotic therapy.

The microbial diversity, determined by the Shannon diversity index, showed a value of 2.37 at baseline versus 2.16 post-therapy. The ACE index, an estimator of the richness of the total quantity of bacteria, was greater at baseline (90) than post-therapy (72). The uniformity (Simpson’s index) was greater post-therapy (3.28) than at baseline (3.37).

### 3.2. Saliva Samples

In the saliva samples, we obtained an average of 84,642 high-quality readings at baseline and 120,246 readings post-therapy, which enabled a complete description of the bacterial diversity in both conditions (Figure 3). We identified a total of 11 phyla, 16 classes, 34 orders, 58 families, 102 genera, and 203 species. There were taxonomic differences between the two samples at the order level, which were even more pronounced at the family, genera, and species levels (Figure 3). After the administration of antibiotics, we observed a relevant reduction in the saliva’s bacterial diversity compared with baseline.

**Figure 5:**
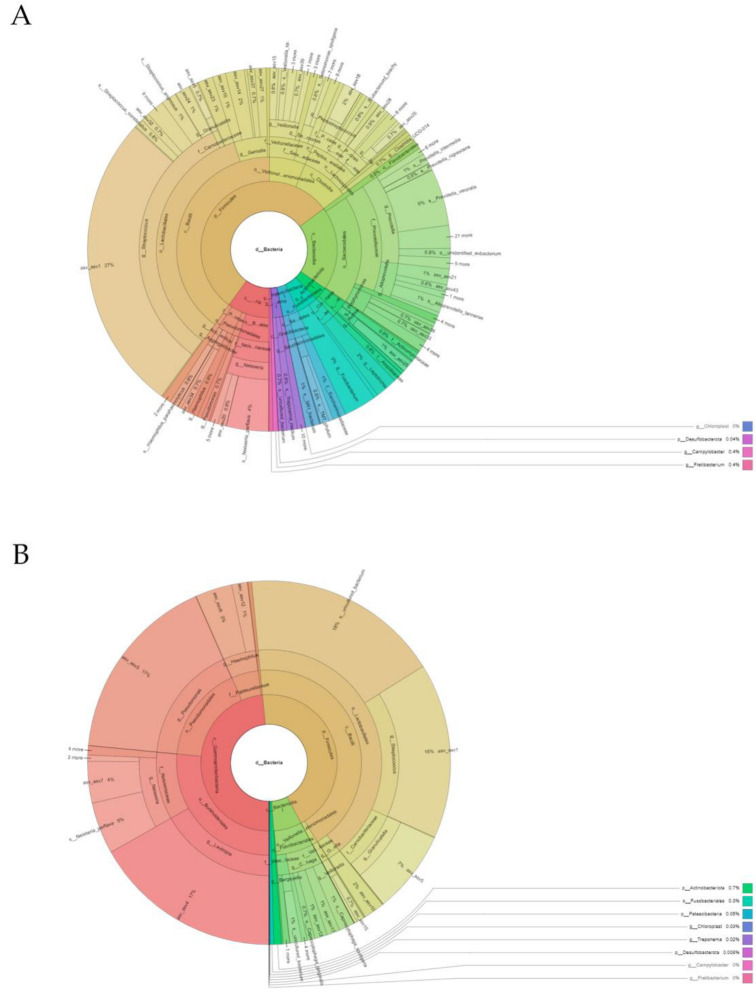
Representation created using the Krona hierarchical browser of the phylotypes identified at the phylum, class, order, family, genus, and species level in an individual with periodontitis: (**A**) Baseline saliva sample; (**B**) Saliva sample after antibiotic therapy.

At baseline, we identified a total of 10 phyla, 15 classes, 32 orders, 54 families, 92 genera, and 186 species. At the phylum level, the most prevalent microbiota were *Proteobacteria* (48.47%), *Firmicutes* (43.77%), and *Bacteroidota* (6.65%) (Figure 5A and Table S3). The most abundant genera (≥5% of relative abundance) were *Abiotrophia* (17.65%), *Pseudomonas* (16.84%), *Lautropia* (16.82%), *Streptococcus* (15.65%), and *Neisseria* (8.85%) (Figure 5A and Table S4).

After completing the antibiotic therapy, the saliva’s microbiota showed a reduction in the relative abundance compared with baseline, with a total of 10 phyla, 11 classes, 23 orders, 39 families, 52 genera, and 73 species. The predominant phyla (≥5% of relative abundance) were *Firmicutes* (54.81%), *Bacteroidota* (19.85%), *Proteobacteria* (9.97%), and *Fusobacteriota* (5.23%) (Figure 5B; Table S3). The most abundant genera (≥5% of relative abundance) were *Streptococcus* (32.74%), *Prevotella* (11.12%), and *Neisseria* (5.20%) (Figure 5B and Table S4).

Of the 102 genera identified in the saliva samples, the relative abundance of 79 genera was considerably reduced post-therapy compared with baseline, and 60% of these became undetectable. As with the subgingival samples, there was a reduction in the abundance of some of the main periodontal Gram-negative pathogens such as *Porphyromonas*, *Treponema*, *Tannerella*, *Prevotella*, *Aggregatibacter,* and *Fusobacterium*, along with other subgingival pathogens such as TM7x and *Alloprevotella*. In contrast, other oral health-related genera such as *Haemophilus*, *Granulicatella,* and *Abiotrophia* showed an increase in their relative abundance post-therapy.

In terms of relative abundance and considering the genera with a presence greater than 1% in at least one of the two situations, of the 12 ASVs identified in the phylum *Bacteroidota*, the genera *Alloprevotella*, *Prevotella,* and *Tannerella* decreased more than 99.0% after administering antibiotics, and the genus *Porphyromonas* was undetectable. In contrast, an increase was detected in the relative abundance of the genera *Chryseobacterium* (79.2%), *Capnocytophaga* (88.1%), and *Bergeyella* (90.7%) (Table S4).

The phylum with the greatest abundance of ASVs in the saliva was *Firmicutes* (specifically 44), approximately 50% of which experienced noteworthy variations in their relative abundance between baseline and post-therapy (Table S4). The genera with a presence greater than 1% that most decreased their relative abundance included *Peptostreptococcus* (99.6%), *Dialister* (99.4%), and *Selenomonas* (99.2%), followed by *Oribacterium* (97.7%), and to a lesser extent *Veillonella* (74.1%), *Gemella* (59.9%), *Streptococcus* (52.2%) and *Mycoplasma* (30.9%). After the antibiotic administration, the genera *Clostridia* UCG-014, *Catonella*, *Centipeda*, *Johnsonella*, *Peptococcus, Peptoanaerobacter*, *Lachnoanaerobaculum*, *Shuttleworthia*, *Parvimonas,* and *Anaeroglobus* became undetectable. In contrast, the genera *Granulicatella* (90.8%) and *Abiotrophia* (99.3%) increased their presence considerably.

For the phylum *Fusobacteriota*, the relative abundance of the three identified ASVs decreased (Table S4), with a post-therapy reduction of 99.6% for the genus *Fusobacterium* and 85.4% for *Leptotrichia* compared with baseline. *Streptobacillus* became undetectable after the antibiotic therapy.

Similarly, of the 7 ASVs identified in the phylum *Patescibacteria*, the post-therapy abundance of the genus *Saccharimonas* decreased by 98.8%. In this situation, the genera TM7x and *Absconditabacteriales*_SR1 were undetectable, although these exceeded 1% at baseline (Table S4).

The antibiotic therapy caused an increase in the relative abundance of the various genera of the phylum *Proteobacteria*, represented by 21 ASVs (Table S4), which included the gamma-proteobacteria *Lautropia* (99.8%), *Pseudomonas* (96.1%), *Haemophilus* (80.1%) and *Neisseria* (41.3%). Additionally, we identified the genera *Eikenella*, *Kingella,* and *Serratia*, which were not detected at baseline. In contrast, this phylum decreased substantially post-therapy in terms of the abundance of the genera *Aggregatibacter* (94.1%) and *Delftia* (93.45%), and the genera *Cardiobacterium, Actinobacillus, Faucicola, Moraxella* and *Stenotrophomonas* became undetectable.

The relative abundance of the genus *Treponema* (the only representative of the phylum *Spirochaetota*) decreased by 99.3% after the treatment, and *Fretibacterium* (the only representative of the phylum *Synergitota*) became undetectable (Table S4).

The microbial diversity, determined by the Shannon diversity index, reached a value of 1.7 at baseline versus 3.2 post-therapy. The ACE index, an estimator of the richness of the total quantity of bacteria, was greater at baseline (92) than post-therapy (52). The uniformity (Simpson’s index) was greater post-therapy (2.27) than at baseline (2.98).

### 3.3. Sample Analysis by Clusters

**Figure 6:**
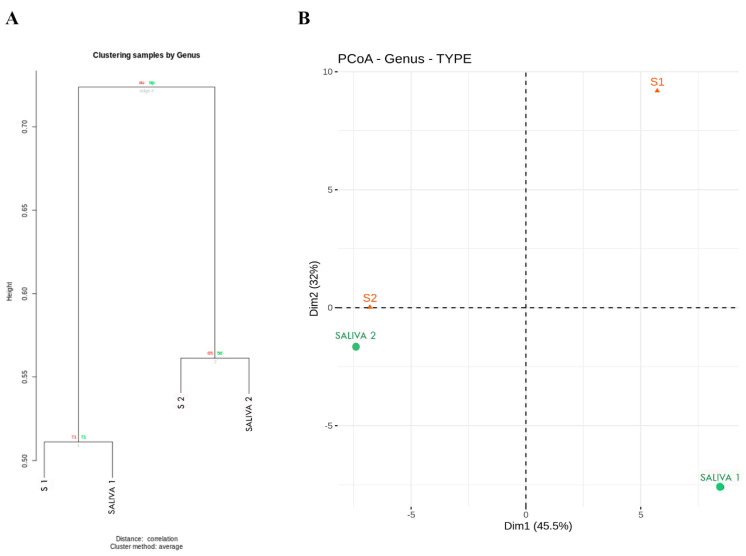
Characteristics of the clusters of samples: (**A**) Identified clusters of the taxa of the saliva and subgingival plaque samples at baseline and post-therapy; (**B**) principal coordinates analysis (PCoA) with weighted UniFrac distances of the samples by cluster assignment. S1, baseline subgingival sample; S2, post-therapy subgingival sample; Saliva 1, baseline saliva sample; Saliva 2, post-therapy saliva sample.

To investigate the relationship between the saliva and subgingival plaque samples before and after the antibiotic therapy, we performed a cluster analysis (Figure 6A). A clear pattern emerged in which the samples were grouped in two clusters, one corresponding to the pretreatment samples, both plaque and saliva, and the other to the post-therapy samples. To evaluate the similarity in bacterial composition among the 4 samples (2 of saliva and 2 of subgingival plaque), we conducted a PCoA (Figure 6B). The UniFrac distance confirmed the difference in the bacterial composition of the 4 samples at the phylum, family, and genus levels.

## 4. Discussion

At the beginning of the case study, a differential diagnosis was made between possible syndromes involving the oral cavity [20,21,22,23]. This patient’s characteristic phenotype and clinical findings enabled a suspected diagnosis of CFCS to be established, which ultimately was confirmed with the genetic study [9]. In the context of RASopathies, CFCS has several manifestations that can determine its dental management such as heart defects, seizures, and intellectual disability. Although this patient presented no congenital heart disease, heart problems in certain cases can appear later (e.g., hypertrophic cardiomyopathy) [24]. Neurodevelopment disorders and particularly intellectual disabilities have a prevalence greater than 80% among patients with CFCS [25], which can limit, as in the present case, the patient’s level of cooperation in the dental setting.

The craniofacial phenotype of RASopathies is so peculiar that it allows a suspected diagnosis to be established in utero [11]. Despite these facial dysmorphic signs, the literature has little information on the oral manifestations of RASopathies, which are highly heterogeneous and are based on case reports [10]. In particular and in regard to periodontal disease, we have found only one study, which included two Mexican patients diagnosed with Noonan syndrome [26]. CFCS is not mentioned explicitly in the reporting of systemic disorders that have a major impact on the loss of periodontal tissues due to their influence on periodontal inflammation [8]. It is not easy to explain the etiopathogenesis of the periodontitis in this patient, given that major immune disturbances have not been detected in RASopathies [27], although autoimmune diseases are particularly prevalent [28]. This patient’s periodontitis can probably be attributed to osteoporosis, a common finding in CFCS [29], which has been related to a greater risk of loss of periodontal attachment [30]. We cannot rule out that hypothyroidism (diagnosed at 4 years of age) has also contributed to the development of periodontal disease [31].

The systemic administration of antibiotics to patients with chronic periodontitis has been mainly employed as an adjunct to scaling and root planning, and the best clinical results are obtained with the combination of amoxicillin and metronidazole [4]. This combination of antibiotics is especially effective in terms of clinical attachment level gain and probing depth reduction in patients with aggressive periodontitis [5,32]. The systemic disorders that have a major impact on the loss of periodontal tissues by influencing periodontal inflammation include neutropenia [8]. In more than 85% of patients with neutropenia, an improvement was observed in the periodontal condition with the adjuvant administration of systemic antibiotics [33]. The literature has few references on the administration of amoxicillin plus metronidazole as the only therapy in patients with chronic periodontitis. In clinical terms (bleeding on probing, probing depth, and attachment level), its efficacy was comparable to that of scaling and root planning [34,35].

It has been shown that the combination of amoxicillin and metronidazole is more effective in microbiological terms than amoxicillin or metronidazole alone and more effective than other antibiotics such as azithromycin and doxycycline [6]. In patients with stage III or IV periodontitis, the adjuvant administration of amoxicillin and metronidazole was more effective than mechanical treatment alone in reducing the levels of several periodontal pathogens and particularly of *Aggregatibacter actinomycetemcomitans* [36]. A clinical trial with a low number of participants with chronic periodontitis assessed the impact (on 40 subgingival species) of amoxicillin plus metronidazole as the only therapy versus scaling and root planning [34]. The authors showed that the total bacterial load and that of most of the bacterial species tested decreased considerably with both treatment modalities and that the baseline bacterial counts had not been recovered 12 months later.

The change in the symbiotic equilibrium between the oral microbial community and the host (dysbiosis) has been incorporated into a new model of periodontal disease pathogenesis [37], which is currently considered the result of a synergistic action of dysbiotic microbial communities [38]. Studies have confirmed that there are substantial differences in the number and composition of bacterial communities (PCoA and UniFrac distance), both in subgingival and saliva samples from individuals with good oral health and those with periodontitis [39,40]. The abundance at the phylum level of *Spirochaetota* (mainly *Treponema*)*, Bacteroidota,* and *Firmicutes* in the patient’s subgingival samples coincides with the findings of other authors in individuals with chronic periodontitis [39,40]. Before the treatment, the abundance of genera classically related to aggressive periodontal diseases such as *Porphyromonas* and *Tannerella* was confirmed [41]. However, we did not detect a notable abundance of periodontal pathogens such as *Aggregatibacter* (especially prevalent in patients with Down syndrome and periodontal disease) [42] or of other genera such as *Filifactor* and *Desulfobulbus* recently related to aggressive periodontitis [41].

The results of the 16S rRNA sequencing, both of subgingival and saliva samples, agreed with those of previous studies that administered adjuvant antibiotics, with more bacterial communities at all phylogenetic levels before the antibiotic therapy, as well as substantial differences in the composition of the communities (PCoA) before and after antibiotic therapy [43]. The administration of amoxicillin plus metronidazole resulted in a significant reduction in the relative abundance of classical periodontal pathogens such as *Porphyromonas*, *Treponema*, *Aggregatibacter,* and *TM7* and an increase in the genera related to periodontal health such as *Actinomyces* (in subgingival plaque samples) and *Streptococcus* (in saliva samples), findings that agree with those reported by other authors in patients who underwent adjuvant antibiotic therapy [44,45].

Studies have suggested that the effects of antibiotics in treating periodontitis last up to 6–12 months, although their clinical impact is longer [34,46]. The analysis of subgingival samples using qPCR has confirmed that the pathogens of the periodontal pocket are not completely eradicated, and therefore reinfection cannot be prevented [47]. The administration of antibiotics alone or as adjuvants to periodontal treatment is determined by the onset of side effects and the development of resistances [36]. The prevalence of side effects (e.g., diarrhea and metallic taste) from the combination of amoxicillin and metronidazole varies significantly, although individuals with diabetes, those younger than 50 years, and women are especially susceptible [48]. The main periodontal pathogenic bacterial species (i.e., *A. actinomycetemcomitans*, *Tannerella forsythia,* and *Porphyromonas gingivalis*) are generally sensitive to amoxicillin; however, some can be metronidazole-resistant (pretreatment). The administration of antibiotics increases the proportion of resistant strains, but this percentage decreases rapidly upon completing the treatment and returns to baseline levels [49]. Accordingly, antibiotic therapy should be reserved for selected cases of aggressive and refractory periodontal disease [50], such as in the present case, in which a new indication is also defined, which is the lack of patient cooperation in performing conventional mechanical periodontal treatment.

## 5. Conclusions

Periodontitis exacerbations as a direct manifestation of systemic disease, presenting a severe clinical expression, can occasionally be controlled exclusively with systemic antibiotics without the need for mechanical periodontal treatment, resulting in substantial changes in the oral microbiome that lead to the recovery of eubiosis of the microbiota.

## Data Availability

The data are available upon request to the corresponding author.

## Supplementary Materials

The following supporting information can be downloaded at: https://www.mdpi.com/article/10.3390/antibiotics11121754/s1, Table S1: Sequenced operational taxonomic units and their proportion (%) in the subgingival samples of a patient with periodontitis at baseline and after antibiotic therapy, for the taxonomic level of phylum; Table S2: Sequenced operational taxonomic units and their proportion (%) in the subgingival samples of a patient with periodontitis at baseline and after antibiotic therapy, for the taxonomic level of genus; Table S3: Sequenced operational taxonomic units and their proportion (%) in the saliva samples of a patient with periodontitis at baseline and after antibiotic therapy, for the taxonomic level of phylum; Table S4: Sequenced operational taxonomic units and their proportion (%) in the saliva samples of a patient with periodontitis at baseline and after antibiotic therapy, for the taxonomic level of genus.
