# Supplementary material for: Analysis of the Oral Microbiome in a Patient with Cardiofaciocutaneous Syndrome and Severe Periodontal Disease: Impact of Systemic Antibiotic Therapy
Source: Antibiotics (Basel). 2022 Dec 4;11(12):1754. doi: 10.3390/antibiotics11121754 (PMC9774349; doi:10.3390/antibiotics11121754)
Supplement: Supplementary file 1 [file antibiotics-11-01754-s001.zip › antibiotics-1975091-supplementary.pdf]

antibiotics

Case Report

Analysis of the Oral Microbiome in a Patient with Cardiofaciocutaneous Syndrome and Severe Periodontal Disease: Impact of Systemic Antibiotic Therapy

Carolina Muñoz Navarro [1,*], María del Carmen Sánchez Beltrán [2], Carolina Arriagada Vargas [1], Pilar Batalla Vázquez [3], Márcio Diniz Freitas [3,*], Jacobo Limeres Posse [3], Pedro Diz Dios [3] and Eliane García Mato [3]

[1] Special Care Dentistry Postgraduate Training Program, School of Medicine and Dentistry, Santiago de Compostela University, 15782 A Coruña, Spain
[2] GINTRAMIS Research Group (Translational Research Group on Microbiota and Health), Department of Medicine, Faculty of Medicine, University Complutense, 28040 Madrid, Spain
[3] Medical-Surgical Dentistry Research Group (OMEQUI), Health Research Institute of Santiago de Compostela (IDIS), University of Santiago de Compostela (USC), Santiago de Compostela, 15782 A Coruña, Spain
* Correspondence: caro.almunoz@gmail.com (C.M.N.); marcio.diniz@usc.es (M.D.F.); Tel.: +34-981523582 (M.D.F.)

TABLES

Table S1. Sequenced operational taxonomic units and their proportion (%) in the subgingival samples of a patient with periodontitis at baseline and after antibiotic therapy, for the taxonomic level of phylum.

Phylum	% at baseline	% post-therapy
Actinobacteriota	2.43	14.45
Bacteroidota	28.33	22.95
Campylobacterota	3.65	0.31
Chloroflexi	0.02	0.06
Desulfobacterota	0.06	0.58
Firmicutes	25.64	37.53
Fusobacteriota	16.99	4.43
Patescibacteria	7.00	2.76
Proteobacteria	10.06	14.95
Spirochaetota	4.68	0.87
Synergistota	1.13	1.12

Table S2. Sequenced operational taxonomic units and their proportion (%) in the subgingival samples of a patient with periodontitis at baseline and after antibiotic therapy, for the taxonomic level of genus.

Genus	% at baseline	% post-therapy
Actinomyces	0.25	0.205
Actinomycetaceae_F0332	0.291	13.339
Corynebacterium	0.8	0.047
Rothia	0.023	0
Pseudopropionibacterium	0.022	0.762
Atopobium	0.009	0
Olsenella	1.039	0.093
Bacteroides	0.098	0.147
Phocaeicola	0.066	0.809
Paludibacteraceae_F0058	3.311	0.944
Porphyromonas	1.984	0.385
Prevotellaceae undefined	0.052	0.177
Alloprevotella	4.945	0.308
Prevotella	10.121	7.984
Prevotellaceae	0.02	0.08
Rikenellaceae_RC9_gut_group	0.061	0.491
Tannerella	1.403	0.524
Capnocytophaga	3.882	8.919
Bergeyella	1.3	1.506
Chryseobacterium	0	0.195
Lentimicrobium	1.089	0.477
Campylobacter	3.654	0.308
Flexilinea	0.022	0.058
Desulfobulbus	0	0.039
Desulfovibrio	0.064	0.542
Bulleidia	0.04	1.744
Solobacterium	0.071	0
Lactobacillales_undefined	0.009	0
Abiotrophia	0.049	7.438
Granulicatella	0.129	7.419
Lactobacillus	0.056	0.039
Pediococcus	0.01	0
Streptococcus	6.358	3.544
Mycoplasma	0.017	1.238
Gemella	2.678	0.998
Clostridia_UCG-014	0.434	0
Pseudoramibacter	0.006	0.028
Defluviitaleaceae_UCG-011	0.111	0.005

Lachnospiraceae_undefined	0.054	0
Butyrivibrio	0.068	0
Catonella	0.876	0.034
Howardella	0.008	0.008
Johnsonella	1.205	0.153
Lachnoanaerobaculum	0.726	0.193
Oribacterium	0.36	0.258
Roseburia	0.009	0
Lachnospiraceae_uncultured	0.301	0
Peptococcus	0.64	0.003
Amnipila	0.022	0
Anaerovoracaceae;_Family_XIII_UCG-001	0.009	0.038
Mogibacterium	0	0.016
Eubacterium_brachy_group	0.181	0.171
Eubacterium_nodatum_group	0.01	0.076
Eubacterium_saphenum_group	0.171	0.49
Anaerovoracaceae_uncultured	0.056	0.202
Filifactor	0.206	1.289
Peptoanaerobacter	0.16	0
Peptostreptococcus	1.228	0.675
Eubacterium_yurii_group	0.144	0.01
Parvimonas	0.444	0.864
Selenomonadaceae_undefined	1.459	0
Centipeda	2.651	0.112
Selenomonas	3.874	2.382
Selenomonadaceae_uncultured	0.038	0
Anaeroglobus	0.024	0.014
Dialister	0.447	1.098
Veillonella	0.297	6.991
Fusobacterium	6.227	1.216
Leptotrichia	10.699	3.21
Streptobacillus	0.061	0
Absconditabacteriales_(SR1)	0.414	0
Saccharimonadales_undefined	0.032	0
Candidatus_Saccharimonas	0.298	0
Saccharimonadaceae	0.857	1.373
Saccharimonadaceae_TM7x	3.517	0.696
Saccharimonadales	1.881	0.694
Lautropia	0.044	5.11
Brachymonas	0.135	0.027
Neisseriaceae_undefined	0.026	0.388
Eikenella	0.052	1.837
Kingella	0	0.51

Neisseria	0.453	3.277
Propionivibrio	0.197	0.01
Cardiobacterium	0.212	0.018
Raoultella	0.084	0.114
Pasteurellaceae_undefined	0.117	0
Aggregatibacter	7.82	0.819
Haemophilus	0.016	2.768
Acinetobacter	0.052	0
Moraxella	0.074	0
Pseudomonas	0.756	0.072
Vibrio	0.022	0
Treponema	4.679	0.867
Fretibacterium	1.128	1.124

Table S3. Sequenced operational taxonomic units and their proportion (%) in the saliva samples of a patient with periodontitis at baseline and after antibiotic therapy, for the taxonomic level of phylum.

Phylum	% at baseline	% post-therapy
Actinobacteriota	0.71	3.18
Bacteroidota	6.65	19.85
Campylobacterota	0.00	0.44
Cyanobacteria	0.03	0.00
Desulfobacterota	0.01	0.04
Firmicutes	43.77	54.81
Fusobacteriota	0.30	5.23
Patescibacteria	0.05	3.70
Proteobacteria	48.47	9.97
Spirochaetota	0.02	2.40
Synergistota	0.00	0.36

Table 4. Sequenced operational taxonomic units and their proportion (%) in the saliva samples of a patient with periodontitis at baseline and after antibiotic therapy, for the taxonomic level of genus.

Genus	% at baseline	% post-therapy
Actinomyces	0.171	0.452
Actinomycetaceae_F0332	0.364	0.409
Bifidobacterium	0	0.019
Corynebacterium	0	0.278
Rothia	0.012	1.162
Pseudopropionibacterium	0.159	0.017
Atopobium	0	0.577
Olsenella	0	0.254
Slackia	0	0.012
Bacteroides	0	0.014
Phocaeicola	0.013	0.002
Paludibacteraceae_F0058	0.014	0.284
Porphyromonas	0	2.812
Alloprevotella	0.02	4.242
Prevotella	0.073	11.125
Rikenellaceae_RC9_gut_group	0.002	0.14
Tannerella	0.003	0.254
Capnocytophaga	4.863	0.576
Bergeyella	1083	0.101
Chryseobacterium	0.573	0.119
Lentimicrobium	0.007	0.184
Campylobacter	0	0.441
Chloroplast	0.034	0
Desulfobulbus	0	0.028
Desulfovibrio	0.008	0.014
Bulleidia	0.01	0.052
Erysipelotrichaceae_UCG-006	0	0.012
Solobacterium	0	0.154
Lactobacillales_undefined	0	1.451
Abiotrophia	17.654	0.12
Granulicatella	7.39	0.677
Marinilactibacillus	0.008	0
Lactobacillus	0.062	0
Pediococcus	0.013	0
Streptococcus	15.654	32.742
Mycoplasma	0.078	0.113
Gemella	1.919	4.791
Clostridia_UCG-014	0	0.654

Defluviitaleaceae_UCG-011	0	0.098
Lachnospiraceae_undefined	0	0.019
Butyrivibrio	0	0.038
Catonella	0	1.03
Howardella	0	0.037
Johnsonella	0	0.345
Lachnoanaerobaculum	0	0.303
Oribacterium	0.003	0.13
Shuttleworthia	0	0.107
Stomatobaculum	0	0.059
Lachnospiraceae_uncultured	0	0.061
Peptococcus	0	0.386
Amnipila	0	0.038
Anaerovoracaceae_Family_XIII_UCG-001	0	0.09
Mogibacterium	0	0.038
Eubacterium_brachy_group	0.002	0.846
Eubacterium_nodatum_group	0	0.109
Eubacterium_saphenum_group	0.006	0.431
Anaerovoracaceae_uncultured	0	0.18
Filifactor	0.007	0.404
Peptoanaerobacter	0	0.101
Peptostreptococcus	0.006	1.628
Eubacterium_yurii_group	0	0.108
Parvimonas	0	1.15
Selenomonadaceae_undefined	0	0.369
Centipeda	0	0.461
Selenomonas	0.01	1.237
Anaeroglobus	0	0.21
Dialister	0.002	0.344
Megasphaera	0	0.066
Veillonella	0.94	3.624
Fusobacterium	0.012	2.909
Leptotrichia	0.286	1.954
Streptobacillus	0	0.368
Absconditabacteriales_(SR1)	0	1.249
Gracilibacteria	0	0.061
Saccharimonadales_undefined	0	0.048
Candidatus_Saccharimonas	0.002	0.175
Saccharimonadaceae	0.041	0.5
Saccharimonadaceae_TM7x	0	0.51
Saccharimonadales	0.01	1.159
Lautropia	16.821	0.037
Comamonas	0	0.024

Delftia	0.007	0.107
Neisseriaceae_undefined	0.176	0
Eikenella	0.335	0
Kingella	0.327	0
Neisseria	8.857	5.201
Propionivibrio	0	0.04
Cardiobacterium	0	0.127
Suttonella	0.008	0
Raoultella	0.097	0
Serratia	0.226	0
Pasteurellaceae_undefined	0	0.017
Actinobacillus	0	0.793
Aggregatibacter	0.056	0.956
Haemophilus	4.664	0.93
Acinetobacter	0.044	0.037
Faucicola	0	0.317
Moraxella	0	0.055
Pseudomonas	16.848	0.663
Stenotrophomonas	0	0.666
Treponema	0.017	2402
Fretibacterium	0	0.363
